# Vulnerability of a Small Population of an Arboreal Mammal to Landscape Change Associated With a New Motorway and Drought

**DOI:** 10.1002/ece3.72112

**Published:** 2025-09-16

**Authors:** Ross L. Goldingay, Brendan D. Taylor, Luke Andrews, David Rohweder

**Affiliations:** ^1^ Faculty of Science & Engineering Southern Cross University Lismore New South Wales Australia; ^2^ Sandpiper Ecological Surveys Pty Ltd Alstonville New South Wales Australia

**Keywords:** BACI, climate variability, Nambucca State Forest, Ngambaa Nature Reserve, population monitoring, Yarriabini National Park

## Abstract

Small populations are vulnerable to extinction due to extrinsic factors such as increasing levels of habitat disturbance and isolation, as well as environmental variation. We investigated the response of a small population of a nationally threatened Australian arboreal mammal, the yellow‐bellied glider ( *Petaurus australis* ; 560 g), to the construction of a new motorway, which increased isolation of this population. We contrasted the small population with two larger populations in conservation reserves in the broader region. We conducted surveys in 6 of 10 years at 92 sites across the three forest areas to describe changes in population occupancy. A severe drought occurred in year 6 of our study. The probability of occupancy in year 1 was lower (0.22) in the small population compared to the larger reserve populations (0.52). The drought had a profound influence on all populations, with lower detection leading into the drought, before detection recovered to pre‐drought levels in the reserves. Additional survey effort using audio‐recorders was employed to detect individuals in the small population of this highly vocal species, but none were detected 4 years after the drought, suggesting local extinction had occurred. Whilst motorway construction increased population isolation, it appears the drought was probably the most consequential factor given its adverse influence on all populations. The three forest areas also contained the coastal sugar glider ( *Petaurus breviceps* ; 100 g), which has much larger population sizes (~28 times larger in the small population area) and a higher reproductive rate compared to the yellow‐bellied glider. Its probability of occupancy (> 0.6) did not differ among the three populations and was unaffected by the drought. The contrasting response in the two species highlights the importance of life history traits when populations are fragmented.

## Graphical abstract

We investigated the response of two species of Australian arboreal mammals, the yellow‐bellied glider and the coastal sugar glider, to the construction of a new motorway. We contrasted these populations with larger populations in two conservation reserves in the broader region over a 10‐year period. The small motorway population of the yellow‐bellied glider appears to have suffered local extinction, primarily driven by a drought in year 6.

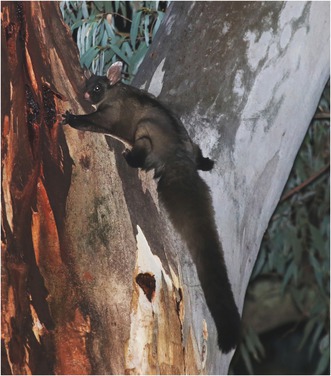

## 1 Introduction

Small populations are known to be at high risk of extinction because they are vulnerable to random demographic and environmental factors (Gilpin and Soulé 1986; Crooks et al. 2001), and to inbreeding depression and loss of genetic diversity (Epps et al. 2005; Frankham 2005). Consequently, conservation management needs to prevent populations from being reduced to low size by attempting to prevent habitat loss and fragmentation (e.g., Beier 1993). Conversely, where this has occurred, management intervention may be able to increase population size over time by increasing available habitat or connectivity to suitable habitat (Hodgson et al. 2011; Lamka and Willoughby 2024).

The construction of roads is a primary cause of habitat loss and fragmentation (Forman et al. 2003; Laurance et al. 2014; Zhang et al. 2015; Engert et al. 2024). Roads not only create barriers to animal movement (Epps et al. 2005; Koivula and Vermeulen 2005; Riley et al. 2006; Eigenbrod et al. 2008), but they directly increase mortality due to vehicle strike (Steen et al. 2006; Ceia‐Hasse et al. 2018) and they may create a zone of road avoidance or reduced abundance (Rytwinski and Fahrig 2012; Andrasi et al. 2021; de Jonge et al. 2022; Ceia‐Hasse et al. 2024), thereby substantially reducing the amount of available habitat in the remnants they create. Thus, roads may inadvertently produce small populations (e.g., Epps et al. 2005; Delaney et al. 2010).

Wildlife road‐crossing structures are now commonly installed along new roads to mitigate the loss of population connectivity and the mortality arising from wildlife‐vehicle strikes (Lesbarrères and Fahrig 2012; Rytwinski et al. 2016; Denneboom et al. 2021; Soanes et al. 2024). Whilst many studies document the use of such structures (e.g., Ng et al. 2004; Mata et al. 2008; Andis et al. 2017; Goldingay et al. 2022; Soanes et al. 2024), there have been few empirical studies conducted at the population level to understand how effective they are (e.g., van der Ree et al. 2009; Soanes et al. 2018; Sawaya et al. 2019). Furthermore, very few studies have employed a before‐after‐control‐impact (BACI) study design to enable unambiguous evaluation of population response to landscape change (see van der Grift et al. 2013; Rytwinski et al. 2015; Soanes et al. 2024). Consequently, there is a need for further empirical studies employing a BACI design to evaluate population responses to new roads.

We investigated the influence of the construction of a four‐lane dual carriageway motorway on a small population of a threatened arboreal mammal, the yellow‐bellied glider ( *Petaurus australis* ; 560 g), in eastern Australia. The construction potentially increased the fragmentation and isolation of the habitat available to this population, requiring the installation of road‐crossing structures (canopy‐bridges and tall wooden poles for gliding), though we do not investigate their effect. Surveys for this species commenced before construction and extended over a 10‐year period. Surveys targeted the small population adjoining the motorway as well as two larger populations in conservation reserves away from the motorway. The primary aim of this study was to evaluate whether there was any discernible influence of the motorway on the small population of the yellow‐bellied glider. The three forest areas also contained a smaller congeneric species, the coastal sugar glider ( *Petaurus breviceps* ; 100 g). This provided an opportunity to investigate a secondary aim, which was to consider whether life history traits influence population resilience to landscape change because the smaller species occurs at a much higher density and has a higher reproductive rate (Quin 1995) compared to the yellow‐bellied glider (Goldingay 2025a). A severe drought occurred in year 6 of our study. This emphasised the importance of including reference populations that are not exposed to landscape change and provided an opportunity to investigate the influence of drought on these two species.

## 2 Methods

### 2.1 Study Species

**FIGURE 1:**
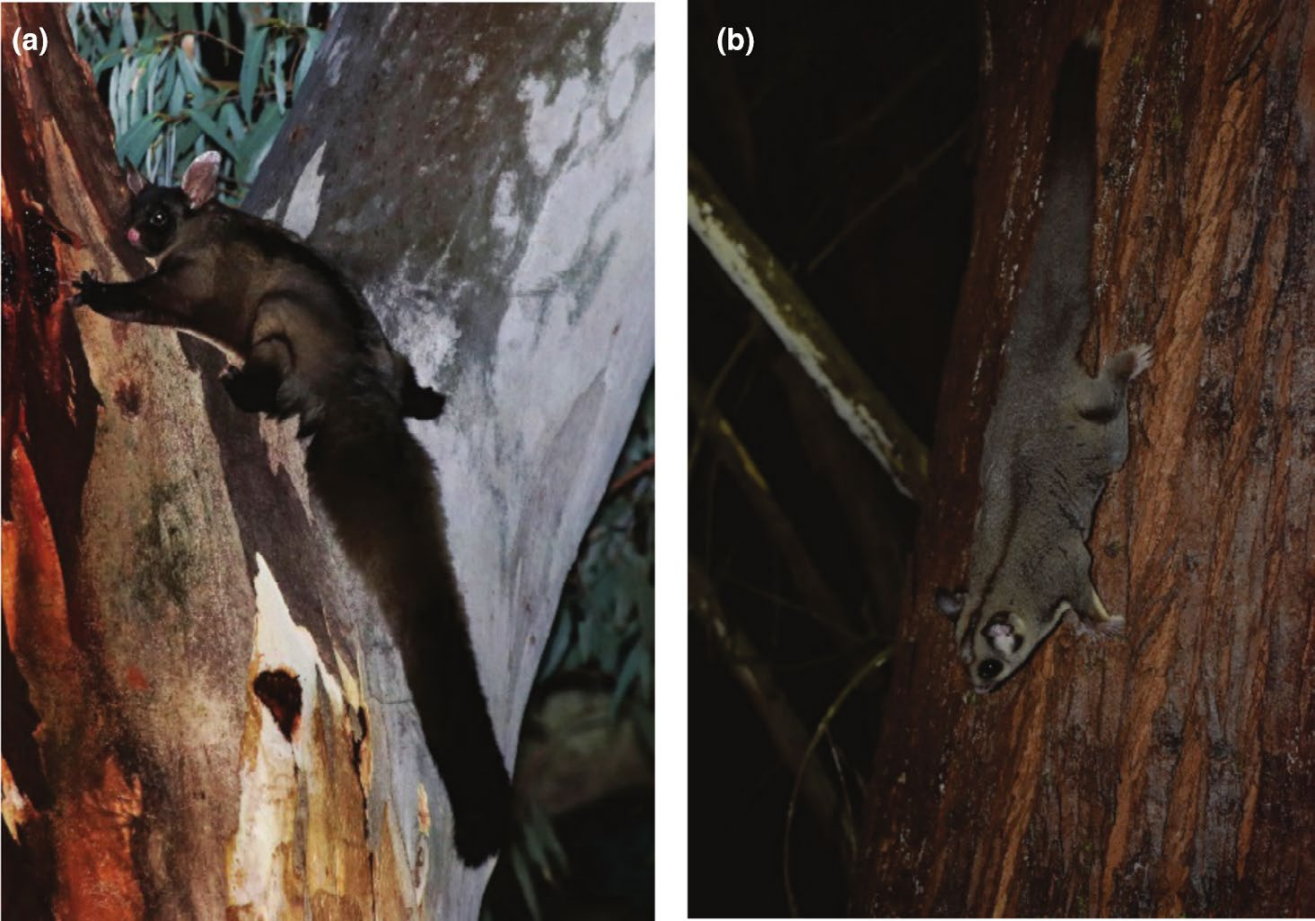
(a) The yellow‐bellied glider. Image: Rohan Bilney. (b) Coastal sugar glider. Image: Ross Goldingay.

The primary focus of this study was the yellow‐bellied glider (Figure 1a), a species listed by the Australian government as vulnerable in 2022 (Department of Agriculture, Water, and the Environment [DAWE] 2022). It is a nocturnal gliding mammal that lives in small family groups of 2–6 individuals that occupy defended territories of 30–80 ha (Goldingay and Kavanagh 1993; Goldingay et al. 2001). In tall forest, it can glide up to 145 m but typically makes glides of 20–30 m when foraging (Goldingay 1989, 2014). Its diet consists predominantly of plant and insect exudates (nectar, sap, honeydew, manna) but also invertebrates (Goldingay 1987, 1990; Kavanagh 1987; Quin et al. 1996; Carthew et al. 1999). On average, it produces < 1 young per year that requires 1–2 years to mature (Henry and Craig 1984; Goldingay and Kavanagh 1990; Goldingay 1992; Goldingay et al. 2001). Its sensitivity to drought was demonstrated by a 48% decline in abundance in one population following a severe drought in 2019 (Goldingay et al. 2023). An additional species for which we had adequate data was the coastal sugar glider ( *Petaurus breviceps* ) (Figure 1a). This species has recently been split into three species (Cremona et al. 2021) with the nominate form in this study now having a restricted geographic range extending along the New South Wales (NSW) coast to the foothills of the Great Dividing Range (Jolly et al. 2023). It also has a diet consisting predominantly of plant and insect exudates and invertebrates (Howard 1989). The coastal sugar glider and its sibling species *P. notatus* live in social groups of 2–7 individuals, produce 1–2 litters of 1–2 young per year, with young reaching sexual maturity in < 1 year of age (Suckling 1984; Quin 1995; Sadler and Ward 1999; Goldingay et al. 2024). The coastal sugar glider occupies home ranges of 2–6 ha (Gracanin and Mikac 2022; McLean et al. 2025). We also detected the broad‐toed feathertail glider (*Acrobates frontalis*; 12 g), another exudivorous species, but too infrequently to include in an analysis.

### 2.2 Study Area

**FIGURE 2:**
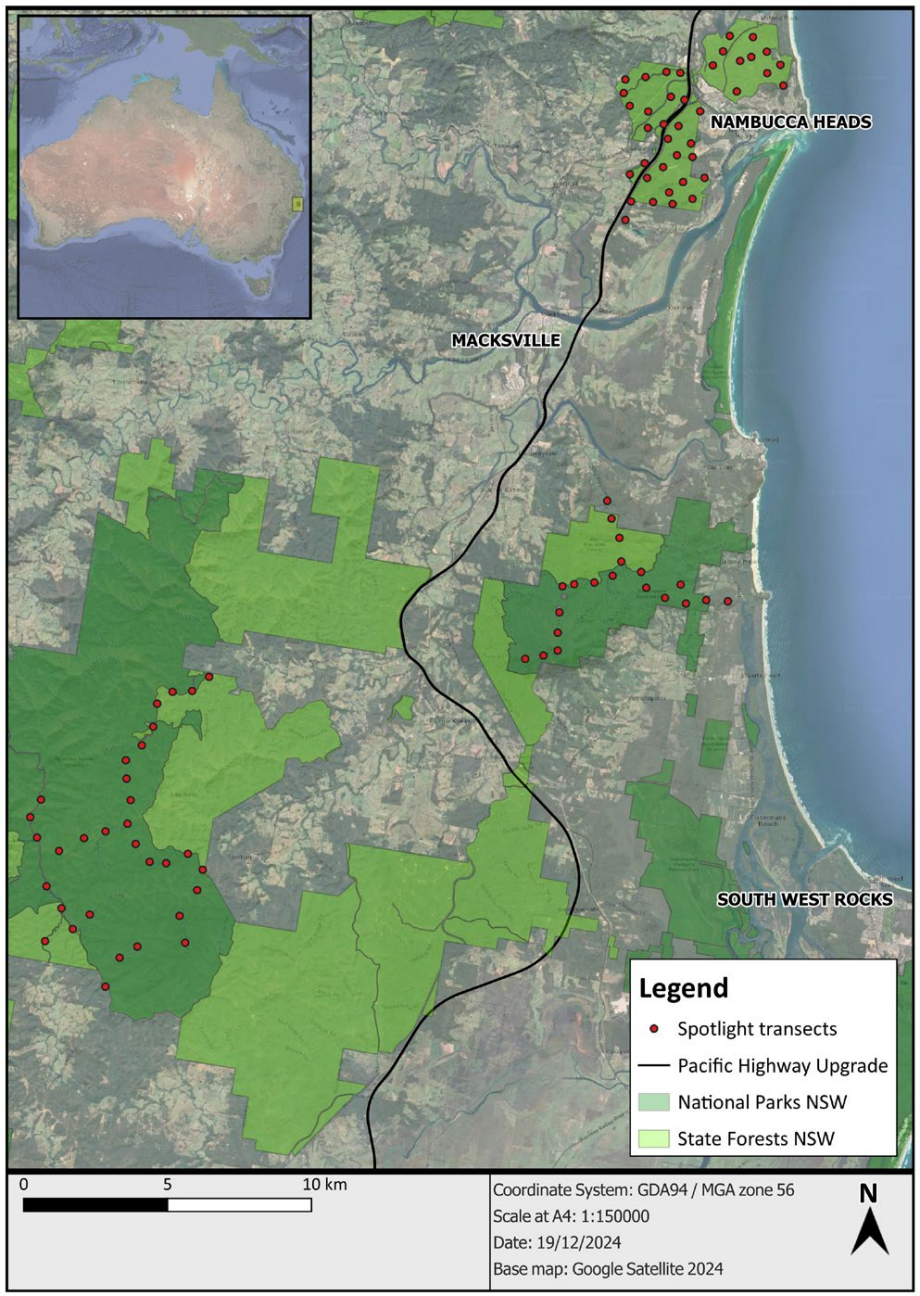
The study area in north‐east New South Wales, Australia and the location of all survey transects across three forest locations. Nambucca forest areas in the north‐east straddle the motorway. Yarriabini is south and Ngambaa is south‐west of Nambucca. The black line shows the new alignment of the Pacific Motorway.

This study was conducted across three forested areas in north‐east NSW, Australia (Figure 2). Nambucca State Forest (hereafter Nambucca; 30°39′7″ S, 152°58′12″ E) in the north, the smallest area, was comprised of three distinct sections, referred to as north‐east (~640 ha), south (~635 ha) and west (~655 ha). Prior to this study, the most recent records of the yellow‐bellied glider (https://atlas.bionet.nsw.gov.au/) in these sections were in 1998 in the north‐east, in 2003 in the south and in 2011 in the west. The north‐east section was essentially isolated from the west section prior to this study due to residential development, the north coast railway and the Pacific Highway. A small local road with a canopy gap of 10–15 m formed the boundary between west and south sections. Construction of the motorway extended from the north in late 2014, further severing the north‐east section. It extended along the boundary between the west and south sections in 2016 and was completed in mid‐2018. The motorway produced canopy gaps of 60–105 m wide that were likely beyond the glide ability of the yellow‐bellied glider. To reduce isolation of the gliders, vegetation was retained in an 850‐m long median strip along the boundary, producing 40‐m wide canopy gaps across one of the two carriageways at ~4 locations. Three rope canopy‐bridges and four sets of glide poles were installed along this section to provide additional connectivity. The rope bridges and poles were not monitored by us or the road agency.

Yarriabini National Park (hereafter Yarriabini; 30°47′29″ S, 152°56′16″ E) is located ~10 km south of Nambucca and encompasses ~2000 ha within a broader forested area of 4400 ha. Ngambaa Nature Reserve (hereafter Ngambaa; 30°51′59″ S, 152°44′60″ E) is located ~17 km south‐west of Nambucca and encompasses 10,500 ha within a broader forested area of 37,000 ha. Forests contained open dry and wet sclerophyll forest dominated by blackbutt (*Eucalytpus pilularis*), tallowwood ( *E. microcorys* ), spotted gum (*Corymbia variegata*), small‐fruited grey gum ( *E. propinqua* ), ironbark ( *E. siderophloia* and *E. fibrosa* ), pink bloodwood ( *C. intermedia* ), flooded gum ( *E. grandis* ) and brushbox ( *Lophostemon confertus* ).

The nearest weather station with temperature data was Southwest Rocks Lighthouse (located 33 km south of Nambucca), which has a mean maximum monthly temperature in the warmest month of 27.1°C (range 24.3°C–30.3°C) and a mean minimum monthly temperature in the coldest month of 11.3°C (range 9.7°C–13.2°C) (Bureau of Meteorology, bom.gov.au). The study area experienced a severe drought in 2019 (year 6 in our study design, see below) at which time Bowraville (located 9 km west of Nambucca) received 46% of its annual average rainfall of 1354 mm, the second driest year in 100 years. Annual rainfall in the 3 years following 2019 was 133%–168% of the average (including the fourth wettest year in 100 years) before declining to 60% of the average in 2023 (year 10 in our study).

### 2.3 Study Design and Survey Sites

The basic design of this study was to compare changes in glider occupancy over time in Nambucca (where the new motorway subdivided sections of forest) with that in two conservation reserves not exposed to landscape change. There were 40 transects established in Nambucca (elevation: < 100 m), 20 transects in Yarriabini (elevation: 80–480 m) and 32 transects in Ngambaa (elevation: 80–280 m) (Figure 2). The higher number within Nambucca was to ensure the response of that population to landscape change was fully documented. Survey transects of 200 m length were established in each of the areas along the main management roads. Transects were placed a minimum of 500 m apart so that yellow‐bellied gliders detected on one transect were from different social groups to those detected on a neighbouring transect, which were surveyed concurrently.

The south section of the Nambucca forest experienced several habitat impacts during the 10‐year study period. In September 2019 an understory and mid‐canopy fire burnt 40 ha of forest and affected part of four survey transects. In 2020 logging (not clear‐felling) occurred over 65 ha. Clearing of 10 ha occurred in 2020 near one site.

### 2.4 Animal Surveys

Surveys commenced approximately 30 min after sunset by a team of 3–4 people. Each transect was spotlighted for a total of 20 person‐min using flashlights typically of 600 lm. Any animals detected were identified. Binoculars were used as required. Most detections of the yellow‐bellied glider are by call, whilst those of the sugar glider are often by call but also by movement and eyeshine (Davey 1990; Goldingay 2021). At the halfway mark, four recorded calls of the yellow‐bellied glider and the powerful owl ( *Ninox strenua* ) were broadcast from a portable speaker, loud enough for yellow‐bellied gliders within at least 200 m of the transect to hear. The powerful owl may occasionally prey on the yellow‐bellied glider, and its call can provoke a vocal response (Irish and Kavanagh 2011; Goldingay et al. 2016). With 2014 as year 1, surveys were conducted in three periods between August and December in each of years 1, 3, 5, 6, 8, and 10 (2023). Repeat surveys within a year were conducted at least 2 weeks apart. Weather conditions were mostly dry and still during surveys, with some occasions of moderate winds and/or light rain. Surveys were conducted to avoid the period around the full moon. Overall, 1931 individual surveys were conducted.

### 2.5 Audio Surveys

The yellow‐bellied glider is a highly vocal species (Kavanagh and Rohan‐Jones 1982; Goldingay, 1994) that can be readily detected by audio‐recording units (Whisson et al. 2021; Goldingay et al. 2023; Gonsalves et al. 2024). We installed Song Meters (Wildlife Acoustics) at various locations within the Nambucca forest to provide additional sampling. In 2014, we installed eight units (SM3) on trees in the southern block; six near (< 300 m) the planned path of the motorway and two > 500 m from it. In 2016, we installed 10 units (SM4); eight as in 2014 and two in the western block (> 700 m from motorway path). Due to the low number of glider groups detected in previous years, we increased the number of SM4s to 18 in 2018; 10 as in 2016 and another four in the west block and six evenly through the north‐east block. This layout was repeated in 2019, 2020, 2021, and 2023. The units operated for the following periods: August–November 2014 (mean 73 nights, range 55–85 nights); August 2016–February 2017 (197, 196–198); October 2018–March 2019 (128, 86–178); August 2019–January 2020 (146, 112–153); August 2020–February 2021 (158, 122–174); August 2021–February 2022 (177, 106–186); August 2023–February 2024 (167, 88–182). The units were programmed to record for 3 h each night, beginning approximately 1 h after sunset to coincide with the period of highest calling activity.

Analysis of the audio recordings during 2014–2018 was by automatic detection using the sound recognition software Song Scope (version 4.0, Wildlife Acoustics). A yellow‐bellied glider call recogniser was built from call recordings. The recogniser may incorrectly identify calls not made by the yellow‐bellied glider (false positives). All audio recordings positively identified by the recogniser were subsequently checked. The recogniser may also fail to identify calls made by yellow‐bellied gliders (false negatives). This could occur due to the remoteness of a calling individual or due to masking by background noise (e.g., heavy rain, calls of other fauna, vehicles). We investigated the rate of false negatives in the 2014 and 2016 recordings. For each site, we randomly selected a 10‐min period on 10 randomly selected nights and searched each period for yellow‐bellied glider calls. Calls were detected in 5 of 180 periods, indicating a false negative rate of 3%. Detected calls likely represent audible calls within approximately 100 m of a recording unit.

By 2020, the Song Scope sound recognition software was superseded by Kaleidoscope Pro (version 5.1.9, Wildlife Acoustics), a more advanced sound recognition software package. Kaleidoscope Pro enabled the development of an advanced classifier for yellow‐bellied glider calls. This was built using calls derived from sound recordings from Nambucca during 2016– 2019. To determine the relative performance of the new recogniser, we analysed seven audio files previously analysed by the Song Scope recogniser and known to contain calls of yellow‐bellied gliders. The new recogniser detected an equal or a greater number of calls than the earlier recogniser on 4 of the 7 sound files. This suggested it was at least marginally better at detecting yellow‐bellied glider calls.

### 2.6 Occupancy Analyses

We employed multi‐season occupancy modelling (MacKenzie et al. 2003) to investigate influences on the annual occupancy dynamics of the study species using the repeat spotlight surveys. The basic model includes four probability parameters that are estimated from the repeat surveys across multiple sites: detection (*p*), site occupancy (psi, *ψ*), site colonisation (gamma, *ɣ*) and site extinction (eps, *ɛ*). Site colonisation and local extinction are estimated based on changes in occupancy after the first primary period (i.e., year) (MacKenzie et al. 2003).

We used programme presence version 15.9 (USGS Patuxent Wildlife Research Centre, Laurel, MD, 20708, USA) to estimate the parameters of detection, occupancy, colonisation, and extinction. Detection histories for each species were constructed from the 21 survey occasions at each site to reflect whether a species was detected (1) or not (0), or if a site was not surveyed (−). Models were constructed to assess site and time‐varying covariates that influenced our study species. The site covariates included: Forest area (Nambucca, Ngambaa, Yarrabini); and Hollows (the number of hollows, entrance > 2 cm diameter) counted in a 0.1‐ha plot at each site in 2014. These covariates were fitted to all parameters. The only time‐varying covariate was year. It was fitted to all parameters except occupancy. Models could be fully year‐varying (i.e., all years different), or partially year‐varying (i.e., some years different and some equivalent). Allowing some years to be different or equivalent is viewed as a plausible scenario due to underlying environmental or biological factors. The estimates from the fully year‐varying models were used to identify different or equivalent years. Reducing the number of parameters in year‐varying models is consistent with the approach of Arnold (2010) to avoid overfitting models.

Akaike's Information Criterion corrected for small sample size (AICc) was used to compare models (Burnham and Anderson 2004). Competing models were ranked from the lowest to highest AICc value. The relative plausibility of a model compared to the top model is suggested by the difference in the AICc value (∆AICc) of the two models. Any model within 2∆AICc of the top model is considered equally plausible. We were guided by Arnold (2010) when adding additional covariates to well‐supported models. If they did not improve model fit by > 2ΔAICc they were viewed as uninformative and excluded. Models that did not converge were excluded. We were guided by Morin et al. (2020) in sequencing our model building. We identified the best performing occupancy covariate and then fitted detection covariates. The top detection models (< 2∆AICc) were retained to again fit occupancy covariates. The top models (< 2∆AICc) then were used to fit covariates to colonisation and extinction. The best fitting model was used to estimate values of occupancy across the 7 years of the study.

Model fit was assessed using presence and employing the method of MacKenzie and Bailey (2004). We fitted the most general single‐season occupancy model and ran 10,000 bootstrap samples. The test statistic suggested the model showed an adequate fit to the data for the yellow‐bellied glider (*p* = 0.08) and the sugar glider (*p* = 0.70).

### 2.7 Population Estimates at Nambucca

We estimated the size of the glider populations for the two forest blocks at Nambucca separated by the motorway, which had a total area of 1290 ha. We used the average probability of occupancy estimated in the years 2014 and 2016 (pre‐drought) to estimate how much of the forest was occupied. We multiplied this by literature values of density. Density of the yellow‐bellied glider has been estimated at 0.04–0.07 individuals per ha based on detailed tracking of tagged individuals in southern NSW (Goldingay and Kavanagh 1993). We use both values but note that Nambucca appears to offer low‐quality dry sclerophyll forest habitat. Yellow‐bellied gliders usually rely on eucalypt sap for a substantial part of their diet (Wallis and Goldingay 2014; Goldingay 2025a) such as where the density values were estimated, but no active sap trees were identified in Nambucca. For the sugar glider, we used estimates of 0.21–0.54 individuals per ha from a location 130 km south, based on detailed mark‐recapture trapping (Quin 1995).

## 3 Results

### 3.1 General Findings

Yellow‐bellied gliders were detected 125 times on 37% of all transects across all years (Table 1). They were detected on 24% of the Nambucca transects in the forest blocks dissected by the motorway before construction (2014–2016) but only one (3%) after construction (2019–2023). They were never detected in the north‐east Nambucca block. Sugar gliders were detected 296 times on 86% of transects across all years. They were detected on 59% of the transects in the motorway forest blocks before construction and 72% of these after construction. They were also detected on 91% of the transects in the north‐east block. The feathertail glider was detected 67 times on 49% of the transects, including 36% of transects in the north‐east block.

**TABLE 1:** The number of survey sites at each location where detections of yellow‐belied gliders (YBG) and sugar gliders (SG) occurred. Each site was surveyed three times each year except in 2020 when only Nambucca was surveyed. Audio‐units were only installed at Nambucca. There were 8–10 during 2014–16 and 18 thereafter.

Locations (survey sites)	2014	2016	2018	2019	2020	2021	2023
YBG							
Nambucca (40)	5	4	1	0	0	1	0
Audio‐units (8–18)	4	5	6	2	0	1	0
Ngamba (32)	10	8	5	2	—	5	6
Yarriabini (20)	8	5	2	3	—	3	6
SG							
Nambucca (40)	7	15	18	21	11	9	7
Ngamba (32)	16	20	23	13	—	8	13
Yarriabini (20)	6	4	2	2	—	3	7

Audio recording units operated for an average of 73–197 nights per annual period. The number of units increased over time to provide a more comprehensive survey. This included six in the north‐east forest block where no yellow‐bellied glider detections were made in any of the five sample years. In the forest blocks adjoining where the new motorway would go, yellow‐bellied gliders were detected at 50% of the units in 2014 and 2016, with detections on an average of 1.8%–2.4% of nights overall. The number of units with detections declined to 1 in 2021 and none in 2023 (Table 1).

### 3.2 Yellow‐Bellied Glider

**FIGURE 3:**
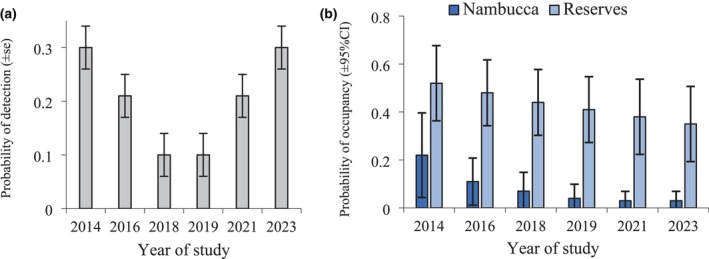
Estimates for the yellow‐bellied glider of (a) the probability of detection and (b) the probability of occupancy. The reserves are Ngambaa and Yarriabini.

There was strong evidence that covariates influenced detection and occupancy, with the top ranked model differing from the null model by 25.71 AICc. There was evidence that initial occupancy differed between Nambucca and the two reserves but not between the two reserves (ΔAICc > 2.0) (Table 2). Detection varied across years; however, there was strong evidence to support the hypothesis that only some years differed (ΔAICc > 4.0). The probability of detection declined substantially during 2014–2019, aligning with the drought, and then increasing through to 2023 (Figure 3a). There was strong evidence that local extinction was higher in Nambucca compared to the reserves (ΔAICc > 7.0). The probability of extinction was estimated per period at 0.53 ± 0.17 for Nambucca and 0.09 ± 0.04 for the reserves. No covariate improved model fit for the colonisation parameter. The probability of colonisation was estimated at 0.01 ± 0.01 per period. The probability of occupancy declined after year 1 but was less marked in the two reserves compared to Nambucca (Figure 3b).

**TABLE 2:** Model selection results (top five models) for the yellow‐bellied glider. Models include parameters for occupancy (psi), colonisation (gamma), local extinction (eps) and detection (p). *W*, model weight; *k*, number of parameters. Covariates: (.), null; N, Nambucca; Ng, Ngambaa; Y, Yarriabini; All‐years, all years different; 3‐years, 3 year‐groups (16, 25, 34).

Model	AICc	∆AICc	*W*	*k*
psi(N), gamma(.), eps(N), p(3‐years)	608.32	0.00	0.97	9
psi(N), gamma(.), eps(.), p(3‐years)	615.77	7.45	0.02	8
psi(N + Ng + Y), gamma(.), eps(.), p(3‐years)	618.01	9.69	0.01	9
psi(N), gamma(.), eps(.), p(all‐years)	620.66	12.34	0.00	10
psi(.), gamma(.), eps(.), p(all‐years)	626.97	18.65	0.00	9

### 3.3 Sugar Glider

**FIGURE 4:**
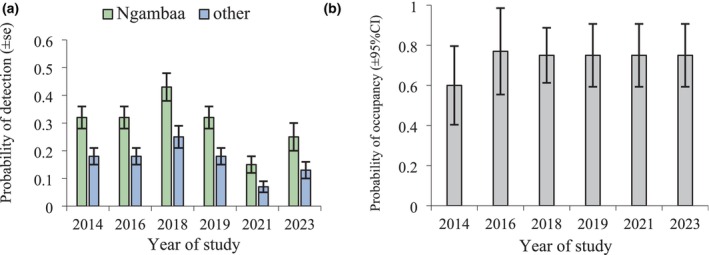
Estimates for the sugar glider of (a) the probability of detection and (b) the probability of occupancy. Other includes Nambucca and Yarriabini.

There was strong evidence that only detection covariates influenced occupancy, with the top ranked model differing from the null model by 39.27 AICc (Table 3). Evidence suggested that no covariates influenced initial occupancy. There was very strong evidence that detection was higher in Ngambaa compared to Nambucca and Yarriabini, which did not differ (ΔAICc > 16.0). Detection varied across years; however, there was strong evidence to support the hypothesis that only some years differed (ΔAICc > 4.0). The probability of detection increased from 2016 to 2018 but dropped substantially in 2021 before increasing in 2023 (Figure 4a). No covariate improved model fit for the colonisation or extinction parameters. The probability of colonisation was estimated at 0.86 ± 0.24 per period and extinction at 0.28 ± 0.08 per period. The probability of occupancy increased from 0.60 after year 1 and remained at ~0.75 (Figure 4b).

**TABLE 3:** Model selection results for the sugar glider. Model parameters as for Table 2. Ng, Ngambaa; All‐years, all years different; 4‐years, 4 year‐groups (124, 3, 5, 6).

Model	AICc	∆AICc	*W*	*k*
psi(.), gamma(.), eps(.), p(4‐years+Ng)	1367.25	0.00	1.00	8
psi(.), gamma(.), eps(.), p(4‐years)	1383.73	16.48	0.00	7
psi(.), gamma(.), eps(.), p(all‐years)	1388.05	20.80	0.00	9
psi(.), gamma(.), eps(.), p(.)	1406.52	39.27	0.00	4

### 3.4 Population Estimates at Nambucca

The mean probability of occupancy in Nambucca was 0.69 in the sugar glider and 0.17 in the yellow‐bellied glider. We estimate a mean population size at the start of the study of 334 sugar gliders (range 187–481) compared to 12 (range 9–15) yellow‐bellied gliders. The latter equates to 5 social groups, based on an average group size of 2.5 individuals (Goldingay et al. 2016). The locations of calling individuals in 2014 and 2016 suggested 6 groups were present. These estimates suggest the population size of the sugar glider was on average ~28 times larger than that of the yellow‐bellied glider.

## 4 Discussion

Arboreal mammals are known to be negatively influenced by roads (McCall et al. 2010; Andrasi et al. 2021; Ceia‐Hasse et al. 2024) but are not commonly the focus of studies designed to investigate their response to new roads. We provide empirical evidence of the response of two species of arboreal mammal to landscape change associated with the development of a new motorway. Importantly, these species have similar diets but differ in body size (560 g vs. 100 g) and consequently differ in spatial requirements and reproductive rates. These attributes produced large differences in population size estimates in the forest blocks adjoining the motorway (12 yellow‐bellied gliders vs. 334 sugar gliders). Such differences have consequences for withstanding disturbances from landscape change and environmental variation (see Soulé et al. 1988; Crooks et al. 2001). Landscape change needs to be evaluated in the context of environmental variation. Our study provides rare insight into the possible mechanism that might drive a small population of an arboreal mammal to local extinction and has lessons for future studies that attempt to evaluate road effects.

### 4.1 Drought as a Population Driver

Droughts are infrequent environmental events that are expected to drive at least some species to lower abundance (e.g., Prugh et al. 2018; Cady et al. 2019; Roberts et al. 2019; Cárdenas et al. 2021). Because droughts occur relatively infrequently, the population response of many species is not well documented. A severe drought occurred mid‐way through our study in 2019. The response of another yellow‐bellied glider population (200 km north) to this same drought has been documented (Goldingay et al. 2023; Goldingay 2025b). At that location, the probability of occupancy remained unchanged over 10 years, but the probability of detection was 50% lower 2 years after the drought year compared to pre‐drought years. The link between detection and abundance was shown by documented site abundance, which was 48% lower 2 years after the drought year compared to pre‐drought. Such contrasts between occupancy and detection reflect that in group‐living species, group size declines before a group's demise (e.g., Goldingay 1992). In the present study, the probability of occupancy showed a steady decline of about 31% over 10 years in the reference populations. Detection declined by > 50% during the drought year relative to pre‐drought but returned to the pre‐drought level 4 years after the drought. The smaller sugar glider showed no change in occupancy but a 50% decline in detection 2 years after the drought year. It maintained high occupancy across the 10‐year study, with no difference between the motorway population and the reference populations. The larger population size and higher reproductive and developmental rates in this smaller species appear to have provided a level of resilience as found in some bird species within habitat islands (Soule et al., 1988; Crooks et al. 2001). This emphasises the importance of considering life history traits when evaluating responses to climate variability (see also Ozgul et al. 2023).

The lower detection in the yellow‐bellied glider in 2018 is a signal that a decline in abundance had commenced prior to the driest year. Annual rainfall in the 2 years before 2014 (first survey year) was close to average (103%–109%). But during 2014–2017 it was below average (62%–91%) and worsened through 2019 (46%). Reduced abundance suggests that breeding was impaired, and mortality had increased leading into the 2018 survey. All three populations had at least 50% fewer detections in 2018 compared to 2014. This may explain the sustained decline since 2014. The insight this provides is that response to environmental variation needs to be understood to be able to determine whether populations are influenced by landscape change.

### 4.2 Landscape Change (Motorway Construction) as a Population Driver

The primary aim of this study was to determine whether a new motorway had a negative effect on the adjoining yellow‐bellied glider population. Our study revealed four insights: (1) this population was very small, consisting of as few as 12 individuals; (2) this population and two reference populations showed declines in abundance associated with a protracted drought that coincided with the completion of motorway construction; (3) no individuals of the motorway population were detected in the final survey year; and (4) the smaller sugar glider with a larger population and a higher reproductive rate was not affected by the motorway.

The key question is whether the small population size and drought led to the apparent demise of the motorway yellow‐bellied glider population or whether the motorway was also responsible? Motorway construction severed the motorway forest blocks in 2015, prior to our second survey in late 2016, potentially isolating 1–2 glider groups in the western block, though glider road‐crossing structures (canopy bridges and glide poles) were installed and retained vegetation provided a small number of potential crossing points. The one glider group with the most exposure to the motorway was detected by audio‐recorder during 2014–2016 in the narrow band of forest on the south‐west side of the motorway. This group was detected in the same area in 2018, 2019, 2020 and 2021 but not 2023, demonstrating it had persisted for 6 years through the disturbance of the motorway. The last spotlighting record at Nambucca was in 2021 of a group located 2 km east of the south‐west group (1.8 km from the motorway) and presumably was a different group. The records for these two social groups suggest the motorway was not the primary driver of the demise of the local population, although some contribution cannot be ruled out. Forest in the south Nambucca section experienced several small‐scale disturbances (fire, logging, clearing) but these should not have been influential due to the large extent of surrounding forest that was unaffected.

Across the 10 years of surveys, the yellow‐bellied glider was never detected in the north‐east section of Nambucca, although there are historic records, whereas the sugar glider was detected at 10 of the 11 sites, including four in the last 2 years. This area had been subjected to intensive logging well before our study commenced. The absence of the yellow‐bellied glider in this area, which has a tenuous connection to the other forest blocks, confirms that small populations of this species are susceptible to landscape and environmental change. Given that the broader Nambucca population had been isolated for many decades, it is plausible that it had lost genetic diversity (e.g., Goldingay et al., 2013), which may have led to inbreeding depression (e.g., Weeks et al. 2017) and may account for an inability to recolonise the north‐east block and to recover from a drought‐induced decline. In contrast, the sugar glider appears to be less susceptible to habitat fragmentation and inbreeding (Gracanin et al. 2023; Knipler et al. 2023).

Differences in life history traits influence species' vulnerability to the negative effects of roads (Rytwinski and Fahrig 2012). For mammals, low reproductive rates, larger body size, and large home range size are useful predictors that a species may be adversely affected by roads (Rytwinski and Fahrig 2011). Our study of two species of exudivorous mammal confirms these predictions; although it appears a drought was probably the most consequential factor. Over a 10‐year period, our monitoring revealed the larger‐bodied (560 g) yellow‐bellied glider, with a low reproductive rate, long maturation time, and small population size, underwent a population decline in the forest adjoining the new motorway, whereas the smaller‐bodied (100 g) sugar glider, with a relatively high reproductive rate, shorter maturation time, and substantially larger population size (~28 times larger), was unaffected. Greater sensitivity to landscape change and drought of one species compared to the other appears to be a consequence of population size and reproductive rate. This is also supported by detections in year 10 at three survey sites in the forest adjoining the new motorway of the tiny and difficult to detect feathertail glider (12 g). It produces 2–8 young per year that mature in < 1 year (Ward 1990). Both the sugar glider and the feathertail glider were detected by camera traps (attached 5‐m high in trees and installed only in year 9) along the vegetated median strip retained between the motorway lanes at Nambucca (Rohweder, D., Taylor, B., and Andrews, L., unpublished data). This suggests this form of mitigation was effective for these species.

### 4.3 Management Implications

There are two important implications from our study. Firstly, the importance of a study design that contrasts an impacted population with reference populations is highlighted. Rainfall variation is an important driver of population dynamics (e.g., Cady et al. 2019) and needs to be anticipated. It was unexpected that a severe drought would coincide with the completion of the motorway construction in our study, with the potential to confound our evaluation of population responses to the motorway. However, monitoring of two independent reference populations allowed us to disentangle a marked drought effect. Our monitoring started one year before construction commenced (i.e., a before‐after‐control‐impact design). Extending pre‐impact monitoring to additional years would be beneficial. Secondly, we were able to contrast two congeneric species that differ markedly in spatial requirements, abundance, and reproductive rate. This revealed that these life history traits have important consequences for coping with environmental variation when populations are confined within relatively large habitat patches of 600 ha. It highlights that we should be concerned for small populations created by roads (e.g., Crooks et al. 2001; Delaney et al. 2010); in our case, one that may have been weakened by forestry activities. Furthermore, there is an obvious need for the appraisal of those populations of threatened species that we cannot afford to lose before future roads fragment landscapes (e.g., Lunney et al. 2022; Gracanin and Mikac 2023; Ceia‐Hasse et al. 2024) and increase vulnerability to climate variability.

## Author Contributions

**Ross L. Goldingay:** conceptualization (lead), data curation (equal), formal analysis (lead), funding acquisition (equal), investigation (equal), methodology (lead), project administration (equal), resources (equal), software (equal), supervision (equal), validation (equal), visualization (equal), writing – original draft (lead), writing – review and editing (lead). **Brendan D. Taylor:** data curation (equal), investigation (equal), methodology (equal), project administration (equal), software (equal), supervision (equal), validation (equal), writing – review and editing (supporting). **Luke Andrews:** data curation (equal), investigation (supporting), project administration (supporting), writing – review and editing (supporting). **David Rohweder:** data curation (equal), funding acquisition (equal), investigation (equal), methodology (equal), project administration (equal), resources (equal), software (equal), supervision (equal), validation (equal), visualization (equal), writing – review and editing (equal).

## Conflicts of Interest

David Rohweder received funding from Transport for New South Wales to conduct this study. Ross Goldingay received funding from this agency in year 1. Other authors declare no financial interests.

## Data Availability

Data used for analysis in this study are accessible at Dryad (https://doi.org/10.5061/dryad.jwstqjqnk).
